# Action Observation Treatment Improves Upper Limb Motor Functions in Children with Cerebral Palsy: A Combined Clinical and Brain Imaging Study

**DOI:** 10.1155/2018/4843985

**Published:** 2018-07-04

**Authors:** Giovanni Buccino, Anna Molinaro, Claudia Ambrosi, Daniele Arisi, Lorella Mascaro, Chiara Pinardi, Andrea Rossi, Roberto Gasparotti, Elisa Fazzi, Jessica Galli

**Affiliations:** ^1^Department of Medical and Surgical Sciences, University of Magna Graecia, Catanzaro, Italy; ^2^Unit of Child Neurology and Psychiatry, ASST Spedali Civili, Brescia, Italy; ^3^Department of Clinical and Experimental Sciences, University of Brescia, Brescia, Italy; ^4^Department of Diagnostic Imaging, Neuroradiology Unit, University of Brescia, Brescia, Italy; ^5^Department of Paediatrics, Ospedale di Cremona, Cremona, Italy; ^6^Department of Diagnostic Imaging, Medical Physics Unit, ASST Spedali Civili, Brescia, Italy; ^7^Neuroscience Unit, Department of Medicine and Surgery, University of Parma, Parma, Italy; ^8^Unit of Child Neurology and Psychiatry, ASST Spedali Civili, Brescia, Italy

## Abstract

The aim of the present study was to assess the role of action observation treatment (AOT) in the rehabilitation of upper limb motor functions in children with cerebral palsy. We carried out a two-group, parallel randomized controlled trial. Eighteen children (aged 5–11 yr) entered the study: 11 were treated children, and 7 served as controls. Outcome measures were scores on two functional scales: Melbourne Assessment of Unilateral Upper Limb Function Scale (MUUL) and the Assisting Hand Assessment (AHA). We collected functional scores before treatment (T1), at the end of treatment (T2), and at two months of follow-up (T3). As compared to controls, treated children improved significantly in both scales at T2 and this improvement persisted at T3. AOT has therefore the potential to become a routine rehabilitation practice in children with CP. Twelve out of 18 enrolled children also underwent a functional magnetic resonance study at T1 and T2. As compared to controls, at T2, treated children showed stronger activation in a parieto-premotor circuit for hand-object interactions. These findings support the notion that AOT contributes to reorganize brain circuits subserving the impaired function rather than activating supplementary or vicariating ones.

## 1. Introduction

There is an urgent need in neurorehabilitation of both adults and children of approaches that take into account the progresses of our knowledge in basic neuroscience. These approaches should aim at transferring ideas and facts from basic neuroscience to clinical practice with the final goal to build up tools well-grounded in neurophysiology and to provide a cure for several neurological (and nonneurological) diseases [1, 2]. Such a rehabilitation approach, grounded in basic neuroscience, would also be a model of translational medicine.

The use of such approaches may help to overwhelm a general attitude in neurorehabilitation to focus on ways to circumvent functional deficits, thus leading to a compensation or a reeducation of functions rather than a cure for them through remediation (for a more general discussion on the notion of compensation and remediation, see [3–5]). Although compensation sometimes works and helps patients to recover in daily activities, it does not aim at repairing the neural circuits underlying specific functions through a direct or indirect restoration. Moving to a translational model in neurorehabilitation would imply to plan specific rehabilitative tools aiming at restoring the neural structures whose damage caused the impaired functions or activating supplementary or related pathways, which may perform the original functions. Last, but not least, rehabilitation tools well-grounded in basic neuroscience allow researchers to plan well-designed randomized controlled trials. This in turn allows clinicians and therapists to measure outcomes not only in terms of functional and/or behavioural gains (as it currently happens by means of functional scales) but also in terms of changes in biological parameters, which researchers can test using neurophysiological and brain imaging techniques. There are indeed some approaches in the neurorehabilitation of children that fit these criteria. For example, constraint-induced movement therapy (CIMT) has a well-established neurophysiological basis grounded on the experimental evidence that monkeys can be induced to use a deafferented limb by restricting movements of the unaffected limb over a period of days [6]. CIMT has been widely applied in patients with acute and chronic stroke and in children with cerebral palsy [7]; similarly, HABIT (hand-arm bimanual intensive training) is a highly structured form of bimanual training, whose goal is to improve the quality and quantity of hand use in bimanual tasks in children with hemiplegic CP [7]. Another example is the mirror therapy [8]. In this treatment, a mirror is placed in the patient's midsagittal plane so that he/she can see her unaffected arm/hand as if it were the affected one. This strategy has been proven to be effective to relieve phantom pain in arm amputees as well as in the recovery of upper limb in chronic stroke patients and in children with cerebral palsy [9, 10]. This approach grounds on a neurophysiological mechanism known as mirror mechanism. Based on this mechanism, the observation of actions performed by other individuals recruits in the observer the same areas involved in the actual execution of those same actions [11]. In the case of mirror therapy, patients have the opportunity to look at their own actions performed with the unaffected arm/hand. More recently, we proposed a novel approach in neurorehabilitation known as action observation treatment (for a review, see Buccino [12]). AOT exploits the mirror mechanism in an even more straightforward manner than mirror therapy, because patients observe daily actions performed by other healthy individuals. During one typical session, patients observe a daily action and afterwards execute it in context. So far, this approach has been successfully applied in the rehabilitation of upper limb motor functions in chronic stroke patients, in motor recovery of Parkinson's disease patients, including those presenting with freezing of gait; interestingly, this approach also improved lower limb motor functions in postsurgical orthopaedic patients [13–16]. Pivotal studies were conducted also in children with cerebral palsy [17–19]. AOT is well-grounded in basic neuroscience, thus representing a valid model of translational medicine in the field of neurorehabilitation. Moreover, the results concerning its effectiveness have been collected in randomized controlled studies, thus being an example of evidence-based clinical practice. The present study aimed at assessing whether this novel rehabilitation approach has the potential to improve the functional recovery of children with CP aged 5–11 (primary school cycle in Italy), within a comprehensive rehabilitation program. The focus was on the recovery of upper limb motor functions. We used the same protocol of an earlier pilot study from our group [17]. We also tested whether this approach may lead to neural changes in the brain by means of a functional magnetic resonance study, in which we asked some of the children that entered the study to manipulate complex objects in the scanner. Control condition was the manipulation of a small sphere.

## 2. Methods

### 2.1. Study Design

We used a two-group, parallel randomized controlled trial. Recruitment criteria and methodological procedures were approved by the Ethical Committee of the Hospital of Brescia.

### 2.2. Participants

All children referred to the Centre of Child Neurology and Psychiatry at the Hospital of Brescia with a diagnosis of cerebral palsy (CP) were eligible. Inclusion criteria were the presence of CP confirmed by neuroimaging techniques (MRI), Manual Ability Classification System (MACS) ≤ 4 [20], verbal IQ > 70, age between 5 and 11 (primary school cycle in Italy), absence of major visual and/or auditory deficits, and no antiepileptic treatment. We enrolled a group of 18 children that met the inclusion/exclusion criteria. Before entering the study, the parents of each child gave written informed consent.

### 2.3. Allocation and Assessment

Patients were enrolled by one of the authors (Elisa Fazzi); enrolled children were randomly allocated to the treatment (*n* = 11) or the control group (*n* = 7) by means of a dedicated software. Both children and their parents were blind to group allocation. After randomization, children were evaluated clinically with a neurological examination carried out by two expert child neurologists (Elisa Fazzi, Anna Molinaro), while functional assessment was carried out by a physician blind to treatment allocation, using the Melbourne Assessment of Unilateral Upper Limb Function Scale (MUUL) and the Assisting Hand Assessment (AHA). MUUL consists of 16 items involving reaching, grasping, releasing, and manipulation, specifically developed to measure quality of upper limb motor functions in children with CP aged 5 to 16 [21]. It has been shown to have a good reliability on a sample of 20 children with different severity degrees of CP. AHA is a hand function evaluation instrument that measures and describes how children with an upper limb disability in one hand use his/her affected hand collaboratively with the nonaffected hand in bimanual actions [22]. In the present study, children underwent functional evaluation with MUUL and AHA at three different time points: at baseline (T1), at the end of the treatment (T2), and at two months of follow-up (T3).

### 2.4. Stimuli

We prepared fifteen video clips to be used during AOT in the treatment group, each showing a specific daily action implying the use of the arms/hands, (i.e., grasping an object, using a pencil, and playing with Lego). All recorded actions were chosen among those which are familiar to children in primary school age. We used the same videos as in a previous study from our group [17]. In that study, we report also a complete list of all seen actions. In the videos, these everyday actions, performed both by normal children and adults, were recorded from different perspectives, to make the video clips more interesting and to sustain the attention of children during the rehabilitation sessions. Each action was subdivided into 3 or 4 constituent motor segments. For instance, eating a candy, one of the shown actions, was subdivided into taking the candy from the table, approaching it to the mouth, and giving back to the therapist. Each motor act was presented for 3 minutes so that the total duration of each video clip was 9–12 minutes. We also prepared the same number of video clips addressing various topics (geography, history, and science adapted for children) but with no motor content, for the control group. Video clips for the control group were also divided into three-four parts, each lasting 3 minutes.

### 2.5. Treatment Procedure

For 3 weeks, children in the treatment group attended daily rehabilitation sessions from Monday to Friday, during which they were asked to observe one movie showing an actor/an actress performing one specific daily action with the hand. Actions were presented in a fixed order according to their complexity, as judged by the experimenter.

After observation of each motor segment (3-4 per each video clip), children were required to execute for 2 minutes what observed to the best of their ability. They were advised that the quality of their imitation was not the goal of the rehabilitation treatment. Children in the control group viewed short video clips (for the same time as treated participants) showing scenes with no motor content (e.g., geographical documentaries). After observing each part of a video clip (3-4 parts per each video clip), controls were also asked to execute the same actions as treated participants for the same duration. In this way, the total amount of visual stimulation and motor activity following observation was similar in the two groups. The only difference concerned the content of videos: treated participants observed videos with motor content (everyday arm/hand actions), while controls observed videos with no specific motor content. As a whole, each rehabilitation session lasted about half an hour. The physiotherapist devoted up to 10 minutes to explain the task and encourage children to observe carefully the videos and perform the seen actions at their best. Twelve minutes was devoted to observation (motor acts for cases, documentaries for controls) and 8 minutes to the execution of the observed actions (cases) or just execution of the same actions, but without a model (controls).

Both treated participants and controls received written instructions. The physiotherapist read them aloud twice. This was in order to avoid any influence of the physiotherapist in giving instructions.

During the treatment, children continued to follow their routine conventional rehabilitation program that was the same for cases and controls. All children (treated participants and controls) completed the study.

### 2.6. Outcome Measures

Primary outcome measures were score changes on the MUUL and AHA.

### 2.7. Statistical Analysis

A mixed linear model, with fixed effects: time (T1, T2, and T3) and group (treatment, control), was carried out on MUUL and AHA scores. The best model was identified using the Akaike information criterion (AIC). The significance level was set at 0.05. Statistical analyses were carried out using SPSS version 23.

### 2.8. fMRI Study

**Figure 1:**
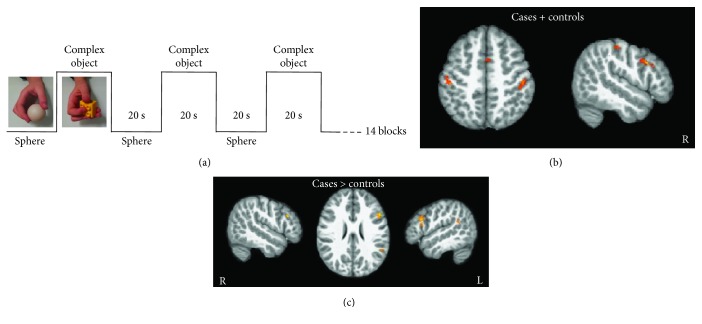
(a) Graphic representation of the fMRI experimental paradigm, alternating manipulation of a simple object (a sphere), and manipulation of complex objects. (b) Clusters of activations transposed on sections from standard pediatric brain (ANTS) before treatment (T1), when comparing manipulation of complex objects versus manipulation of a sphere. Cases and controls are taken as a whole group, *p* < 0.001. Note that at T1, no activation was present when directly comparing cases versus controls. (c) After treatment (T2), direct comparison between cases and controls shows increased activations in frontal and parietal areas known to be involved in hand-object interactions, *p* < 0.001. Clusters of activations transposed on sections from standard pediatric brain (ANTS), as in (b).

Twelve children (six treated participants) out of 18 enrolled children also entered an fMRI study to assess a reorganization of brain neural structures following treatment. While being scanned, children with CP from both groups manipulated complex objects with both hands, in order to explore all the motor properties of the manipulated object. As a control condition, children manipulated a simple object, a sphere. All objects used in the scanner were different from those used during the treatment.  Figure 1(a) shows the experimental paradigm. fMRI data were collected on a 1.5T Siemens Avanto scanner. The protocol included four EPI sequences (TR/TE 2500/50 ms, 3.3 × 3.3 × 3.3 mm isotropic voxel) and a high resolution T1W 3D MP-RAGE sequence for anatomical reference (TR/TE 2050/2.56 ms, 1 × 1 × 1 mm isotropic voxel). Imaging data were collected before starting treatment (T1) and at the end of treatment (T2). The fMRI paradigm consisted of 14 alternating task-rest blocks (8 volumes/block were acquired) repeated 4 times to increase statistics. fMRI data underwent the following preprocessing. The mean EPI was first computed for each participant and visually inspected to ensure that none showed artifacts. The first 2 EPI volumes of each functional run were discarded to allow for T1 equilibration effects. For each subject, all volumes were spatially realigned to the mean volume of the four runs. Next, the 3D structural data of each subject were normalized to the ANTS standard space, a T1 pediatric template in a standardized MNI space [23]. The normalization matrix was subsequently transferred to the fMRI images, resampled in 1 mm × 1 mm × 1 mm voxels using trilinear interpolation in space and then the images were spatially smoothed with a 6 mm full width at half maximum isotropic Gaussian kernel for the group analysis. No participant showed head movements greater than 3 mm; thus, none was excluded from further analyses.

Data were analyzed using a random effects model [24], implemented in a two-level procedure. In the first level, single-subject fMRI data entered an independent general linear model (GLM) by design matrixes modelling the onsets and durations of two experimental factors, one related to the experimental task and one related to its corresponding baseline. For each participant, we generated contrast images displaying the effect of the experimental task (manipulating complex objects) contrasted with the respective baseline (manipulating a sphere). Next, each contrast entered a second level GLM to obtain (i) SPM{T} maps (one sample *t*-test) related to each task at group level and (ii) to test for the existence of brain areas specifically involved in manipulating complex objects. Moreover, we were interested in assessing differences in brain area recruitment between treated children and controls. For all analyses, location of the activation foci was determined in the stereotaxic space of the MNI coordinates system. A significance level of *p* < 0.001 uncorrected and an extended threshold on cluster dimension of 10 voxels was applied.

## 3. Results

Demographic data, clinical features, and brain imaging findings of children in the two groups are shown in Table 1. Mean scores and SD of AHA and MUUL in treated participants and controls at T1, T2, and T3 are shown in Table 2.

Mixed linear model showed that for MUUL, the best model included the random effects of intercept and the fixed effect of group, time, and interaction. For the AHA, the best model included the random effects of intercept and time and the fixed effects of group and interaction. The mixed linear model analysis disclosed a significant interaction between time and treatment, both for MUUL (*b*_1_ interaction = 2.71, *t*_36_ = 3.99, and *p* = 0.000) and for AHA (*b*_1_ interaction = 2.36, *t*_18_ = 3.61, *p* = 0.002). Score improvements, in both scales, were higher in the treated participants than in the controls; furthermore, in the treatment group, those improvements were not only maintained but became even stronger at T3.

**Figure 2:**
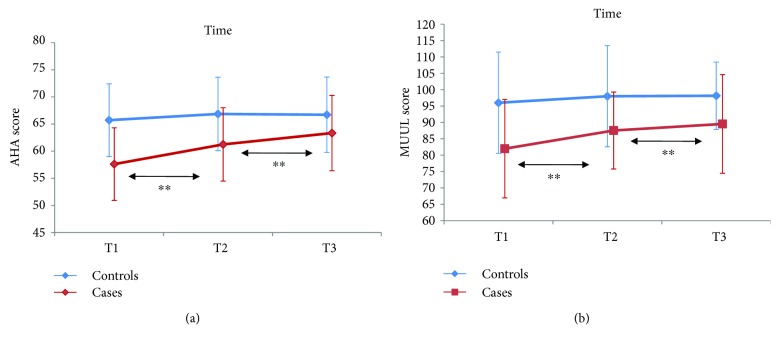
Scores obtained by cases (red line) and controls (blue line) at T1, T2, and T3 in two different functional scales (AHA, MUUL). Statistical analysis (see text for details) showed that only in case scores obtained at T2 differed significantly from scores at T1 in both scales. This was true also when comparing T3 with T2 in both scales (error bars: 95% CI). ^∗∗^refers to statistical significant effects.

Post hoc analysis showed that for MUUL, results at T2 were significantly different from results at T1 only in cases (*p* < 0.001), but not in controls. As for AHA, results at T2 were significantly different from results at T1 (*p* < 0.001). Even more interestingly, results at T3 were different from results at T2 (*p* < 0.001) for both scales, but again only in cases, but not in controls.  Figure 2 shows the results.

## 4. fMRI Results

For the aim of the present study, we will present results concerning the differences between treated children and controls. It is worth stressing that at baseline (T1), there were no differential activations when comparing cases versus controls. In contrast, after treatment (T2), differential activations were located in the left premotor cortex extending to the inferior frontal gyrus (−49; 19; 26), in the right premotor cortex (53; 14; 31), in the left supramarginal gyrus (−47; −51; 37), and finally a weaker activation in the left superior temporal gyrus (−52; −47; 23). Figures 1(a) and 1(b) shows fMRI findings.

## 5. Discussion

The results of the present study are relevant within the literature devoted to rehabilitation of children with cerebral palsy. Treated children improved significantly as compared to controls in both MUUL and AHA. These results are in keeping with earlier, pilot studies using AOT as a rehabilitation tool [17, 18]. It is worth stressing that our sample consisted of hemiplegic (both right and left) and tetraplegic children, thus suggesting that AOT may be useful for different clinical presentations of CP. As reported above, AOT exploits a neurophysiological mechanism known as mirror mechanism. The observation of actions performed by other individuals recruits in the observer the same areas involved in the actual execution of those same actions [11], this whatever the biological effector involved in the observed action. This mechanism may underlie the capacity to understand and imitate others' actions even at an early stage of life [25, 26] and contribute to interact with other people in an empathic manner (for review see, Hari and Kujala) [27]. This same mechanism may be helpful during learning motor tasks or relearning daily actions following brain lesions and therefore during rehabilitation [28–30]. AOT has the potential to become a routine approach in the rehabilitation of children with CP and could be easily applied by physiotherapists working with children. During the rehabilitation session, physiotherapists have the role to motivate little patients to observe carefully every detail of the observed actions and to push children to use the objects provided at hand, as in the videos, but also to reassure if children fail in performing the observed actions. Patients, even children as in the present study, may follow the rehabilitation program without difficulties. It is worth stressing that AOT may be applied in a very flexible manner: in fact, the trained actions, presented through videos, may vary depending on the real need of patients. For example, children that have more difficulties in performing distal hand/arm actions (i.e., grasping, manipulating) should focus their training on these motor tasks, while children that present with impairment of proximal arm actions (i.e., reaching objects, coding objects in space) should train this kind of motor tasks. Last, but not least, AOT may be used also at home where children may get their rehabilitation session with the help of their parents or even in telerehabilitation with a physiotherapist monitoring from a dedicated position what children perform at home.

In the present study, we collected functional scores on MUUL and AHA also at two months of follow-up. Interestingly, treated children, as compared to controls, maintained and even improved their functional gain at follow-up. In our opinion, these findings may be explained by the fact that during AOT, children learn novel strategies to interact with other people and common objects. They learn to look very carefully at all details present in the scene, they pay attention at the different motor segments of an action, and they spontaneously prepare themselves to imitate a seen action or to interact upon objects available in the environment. Eventually, they transfer these strategies in everyday life situations; thus at the very end, they accomplish the goal of gaining better motor performances.

A main point of interest in the present study is that some of the treated children also underwent an fMRI study aimed at assessing whether AOT may recruit areas within the motor system and eventually contribute to their reorganization. It is worth noting that, while being scanned, children performed an independent task, namely, manipulation of complex objects that were not included in the set of actions trained during the treatment. When comparing treated participants and controls, differential activation was present in a sector of the premotor cortex and parietal cortex also involved in object manipulation in both healthy adults and children [31, 32] and known to be endowed with a motor representation of distal upper limb movements. This premotor sector is strictly connected with a parietal area with which it builds up a sensorimotor circuit allowing individuals to code for the motor properties of objects and the implementation of the most appropriate actions to act upon objects [33]. These findings suggest that the brain target of AOT is exactly a hand motor area possibly involved in executing actions as well as in their processing. It therefore appears that there are not vicariating areas emerging from AOT treatment, but rather a recovery of areas normally involved in a specific hand motor task. Further studies should assess to what extent this concerns also other biological effectors (e.g., the foot) and contributes to rebuild physiological sensorimotor circuits. Another issue that future studies should help to ascertain is whether there are specific subgroups of children with cerebral palsy that may mostly benefit from AOT, or rather this approach may help clinical conditions in all children affected.

## Figures and Tables

**Table 1:** Demographic data, clinical features, and radiological findings in treated participants and controls.

Pt. number	Case/control	Sex (M, F)	GA (wk)	Age (yr, m)	CP type Hagberg	Motor abnormalities	GMFCS	MACS	CFCS	Associated impairments	Total IQ	Verbal IQ	Performance IQ	Radiological findings (brain MRI)
1	Case	M	33	9 yr, 5 m	Right hemiplegia	Unilateral spastic hypertonia	2	2	1	V: CVI; H: no; M/A: no; LD: no; E: no	85	92	82	Right temporooccipital, left occipitoparietal, bilateral periventricular, and left frontotemporal subdural hematomas

2	Case	F	27	8 yr, 2 m	Right hemiplegia	Unilateral spastic hypertonia	1	2	1	V: ROP; H: no; M/A: no; LD: no; E: no	100	106	107	Mild bilateral periventricular leukomalacia, mild ventricular dilatation

3	Control	M	40	7 yr, 10 m	Right hemiplegia	Unilateral spastic hypertonia	2	2	1	V: no; H: no; M/A: no; LD: no; E: no	99	101	97	Left subdural occipitotemporal hematoma and epidural parietotemporal hematoma; hypoxic ischemic encephalopathy characterized by signal alterations in both putamen tail and anterior thalamus

4	Control	M	34	8 yr, 3 m	Tetraplegia	Bilateral spastic hypertonia, left side more affected	4	3	2	V: CVI; H: no; M/A: yes; LD: no; E: no	73	97	50	Hypoxic ischemic injury with thinning of the corpus callosum, enlargement of CSF spaces, widespread hyperintensity of centrum semiovale, corona radiata, and periventricular white matter, dilation of the ventricles

5	Control	F	40	6 yr, 8 m	Tetraplegia	Bilateral spastic hypertonia, left side more affected	4	2	3	V: CVI; H: no; M/A: yes; LD: no; E: no	114	139	77	Diffuse periventricular hyperintensity with parietal bilateral white matter involvement; mild dilatation of bilateral ventricular trigone

6	Case	F	30	11 yr, 9 m	Tetraplegia	Bilateral spastic hypertonia, left side more affected	4	3	2	V: CVI; H: no; M/A: yes; LD: yes; E: yes	56	89	50	Periventricular leukomalacia, fronto-parieto-occipital white matter reduction, ex vacuo enlargement of bilateral ventricles

7	Control	F	37	9 yr, 1 m	Right hemiplegia	Unilateral spastic hypertonia	1	2	1	V: CVI; H: no; M/A: yes; LD: no; E: no	87	84	100	Left periventricular malacic area with gliosis, extended into the corona radiata; left corticospinal projection hyperintensisty with mild cerebellar peduncle hypotrophy (Wallerian degeneration)

8	Control	F	31	8 yr, 9 m	Right hemiplegia	Unilateral spastic hypertonia	2	1	1	V: CVI; H: no; M/A: no; LD: no; E: no	87	99	77	Bilateral parietal cystic periventricular leukomalacia, with centrum semiovale white matter involvement, short distance between cortex and ventricular walls in temporoparietal areas, thinning of the corpus callosum

9	Case	F	Not known	11 yr, 9 m	Left hemiplegia	Unilateral spastic hypertonia	2	2	1	V: no; H: no; M/A: no; LD: no; E: yes	Leiter -R 82			Right fronto-parieto-temporal malacic area, ex vacuo enlargement of the ventricle and Wallerian degeneration of the corticospinal tract

10	Case	M	32	6 yr, 10 m	Tetraplegia	Bilateral spastic hypertonia, right side more affected	3	2	2	V: CVI; H: no; M/A: yes; LD: no; E: no	89	120	70	Periventricular leukomalacia, corpus callosum hypoplasia, hippocampal commissure agenesis

11	Control	M	38	5 yr, 2 m	Right hemiplegia	Unilateral spastic hypertonia	2	3	2	V: CVI; H: no; M/A: no; LD: no; E: no	98	118	87	Cortical laminar necrosis (left insular cortex, left frontoparietal areas, and left temporal lobe). Signal T2 and FLAIR hyperintensity in the left caudate nucleus and in the left corona radiata (ischemic event)

12	Case	F	31	10 yr, 1 m	Tetraplegia	Bilateral spastic hypertonia, left side more affected	4	3	3	V: CVI; H: no; M/A: yes; LD: no; E: no	85	92	82	Severe periventricular leukomalacia with major involvement of the posterior area, associated with supra- and subtentorial ventricular dilatation and subarachnoid spaces enlargement, thinning of the corpus callosum

13	Case	F	41	8 yr, 2 m	Right hemiplegia	Unilateral spastic hypertonia	3	2	1	V: CVI; H: no; M/A: no; LD: no; E: yes	87	99	77	Left hemispheric atrophy (previous extensive left frontoparietal intraparenchymal hemorrhage, wide left parietal subdural hematoma), ex vacuo dilatation of the ipsilateral ventricles and midline brain right to left shift, Wallerian degeneration of the corticospinal tract and ipsilateral cerebellar peduncle atrophy

14	Case	M	33	5 yr, 10 m	Right hemiplegia	Unilateral spastic hypertonia	2	3	2	V: strabismus; H: no; M/A: no; LD: no; E: no	85	92	82	Left fronto-parieto-temporo-insular polymicrogyria (perisylvian and perirolandic with cortical infolding), mild left temporal atrophy with subarachnoid spaces enlargement

15	Case	F		6 yr, 8 m	Left hemiplegia	Unilateral spastic hypertonia	4	3	1	V: no; H: no; M/A: yes; LD: no; E: yes	100	106	107	Ischemic right frontoparietal malacic area with focal cortical atrophy, gliosis, subarachnoid space enlargement, and mid ipsilateral ventricular dilatation. Mild controlateral periventricular white matter hyperintensity

16	Case	M	38	6 yr, 3 m	Left hemiplegia	Unilateral spastic hypertonia	2	3	2	V: no; H: no; M/A: no; LD: no; E: no	99	101	97	Malacic areas affecting the right middle cerebral artery territory with Wallerian degeneration of the corticospinal tract and of the thalamus, left hemisphere hypotrophy

18	Case	M	40	5 yr, 3 m	Left hemiplegia	Unilateral spastic hypertonia	2	3	1	V: strabismus; H: no; M/A: no; LD: no; E: yes	101	106	100	Right periventricular porencephalic lesion (hemorrhagic venous infarct) with hemosiderin deposition and Wallerian degeneration of the ipsilateral corticospinal tract

18	Control	M	36	6 yr, 4 m	Left hemiplegia	Unilateral spastic hypertonia	1	2	1	V: no; H: no; M/A: no; LD: no; E: no	90	94	93	Supratentorial right malacic areas with right lateral ventricular dilatation; hemosiderin deposition secondary to germinal matrix hemorrhage

M: male; F: female; GA: gestational age; CP: cerebral palsy; GMFCS: Gross Motor Function Classification System; MACS: Manual Ability Classification System; CFCS: Communication Function Classification System; V: vision; CVI: cerebral visual impairment; H: hearing; M/A: memory and attention; LD: learning disabilities (North American usage; mental retardation); E: epilepsy; MRI: magnetic resonance imaging.

**Table 2:** Mean scores (and SD) of AHA and MUUL in controls and treated participants at different time points.

Group	Score	Time point		
		T1	T2	T3
Control	AHA	65.71 (7.23)	66.86 (7.31)	66.71 (7.52)
	MUUL	96.00 (16.73)	98.00 (16.69)	98.14 (16.52)
Treatment	AHA	57.45 (12.18)	61.09 (10.79)	63.18 (11.06)
	MUUL	81.73 (22.38)	87.27 (22.36)	89.27 (22.41)
